# Supplementary material for: The causal role of circulating inflammatory markers in osteoporosis: a bidirectional Mendelian randomized study
Source: Front Immunol. 2024 Jul 18;15:1412298. doi: 10.3389/fimmu.2024.1412298 (PMC11291241; doi:10.3389/fimmu.2024.1412298)
Supplement: Supplementary file 1 [file DataSheet_1.zip › supplementary material/CIM-OSTEOPOROSIS /GCST90274799.csv_forest.pdf]

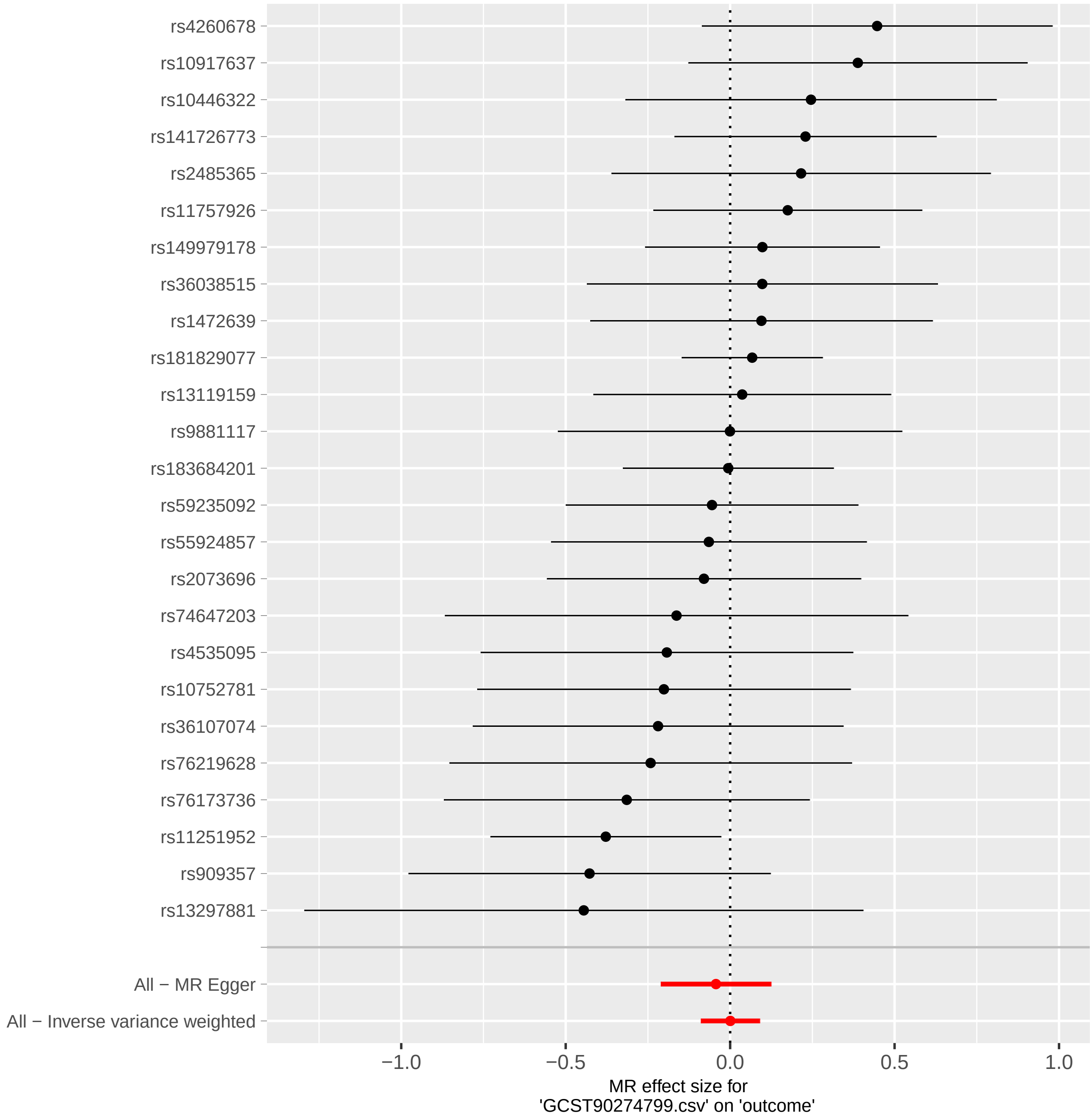
rs4260678
rs10917637
rs10446322
rs141726773
rs2485365
rs11757926
rs149979178
rs36038515
rs1472639
rs181829077
rs13119159
rs9881117
rs183684201
rs59235092
rs55924857
rs2073696
rs74647203
rs4535095
rs10752781
rs36107074
rs76219628
rs76173736
rs11251952
rs909357
rs13297881
All − MR Egger
All − Inverse variance weighted
−1.0
−0.5
0.0
0.5
1.0
MR effect size for
'GCST90274799.csv' on 'outcome'
